# Clinical Analysis of Approach Selection of Extraction of Maxillary Embedded Mesiodens in Children

**DOI:** 10.1155/2022/6517024

**Published:** 2022-05-03

**Authors:** Jie Kong, Zhaowei Peng, Tianhang Zhong, Huang Shu, Ji Wang, Yiyuan Kuang, Guicong Ding

**Affiliations:** ^1^Department of Stomatology, Shenzhen Children's Hospital, Shenzhen 518000, China; ^2^Shuya Dental Clinic, Chengdu 618000, China

## Abstract

**Objective:**

To analyze the relationship between the position of embedded mesiodens in maxilla and surgical approach in children and thus provide reference materials for surgical design.

**Methods:**

According to the preoperative cone-beam computed tomography (CBCT) examination, the location and surgical approach characteristics of 625 children aged 4-16 years old who presented with embedded mesiodens in maxilla and were diagnosed in our department from January 2016 to December 2021 were statistically analyzed.

**Results:**

There were 877 embedded mesiodens in 625 children. The selected cases were classified according to the axial angle relationship between mesiodens and adjacent normal teeth or tooth germs, including 84 cases of acute angle type (including the same direction) (13.4%), 66 cases of vertical type (10.6%), 114 cases of obtuse angle type (18.2%), 271 cases of inverted type (43.4%), and 90 cases of mixed type (14.4%). The palatal gingival margin approach was the most selected surgical approach for the cases of acute angle (including synclastic), obtuse angle, and inverted type, and the palatal gingival margin approach and the combined labial-palatal approach were the most selected surgical approach for the cases of vertical and mixed type.

**Conclusion:**

Palatal gingival margin approach was the most common surgical approach for various types of embedded mesiodens in maxilla in children. Surgeons should classify the case of mesiodens according to the preoperative imaging examination and design the surgical approach reasonably.

## 1. Introduction

Maxillary mesiodens is defined as the presence of supernumerary teeth which locates in the midline area of maxilla [1] and is the most common type of supernumerary teeth, accounting for about 90-98% of total [2–4]. They are frequently found in mixed dentition with an incidence rate of 0.6-1.7% [5], representing a common developmental malformation in pediatric dentistry. Mesiodens are more common in males, with a 1.7 : 1-3.1 : 1 predominance of males to females [6, 7].

Though various theories have been proposed to expound the etiology of mesiodens, there is no definite conclusion yet [1, 4–6, 8, 9]. Mesiodens can affect the normal eruption of adjacent permanent teeth, lead to a variety of malocclusion, and induce odontogenic cysts. Therefore, it is generally considered that they should be removed surgically [10, 11].

Previous studies made classifications of mesiodens according to the characteristics of mesiodens, including the location, crown direction, and morphology [9, 12, 13], but the correlation between classifications and surgical approaches had not been discussed. Our study selected 625 children who underwent extraction of embedded mesiodens in the maxilla as samples, summarized the characteristics of the axial position of mesiodens and the intraoperative surgical approach through preoperative CBCT, and analyzed their internal relationship, with the aim to provide an objective basis for the design of extraction of embedded mesiodens in maxilla.

## 2. Materials and Methods

### 2.1. Case Materials

625 children who were diagnosed as embedded mesiodens in maxilla in our department from January 2016 to December 2021 were analyzed, including 478 males (76.5%) and 147 females (23.5%), with a male to female ratio of about 3.25 : 1. The age of these cases ranged from 4.2 to 16.5 years old, with an average age of (7.53 ± 2.05) years old. There were 168 cases in primary dentition (26.9%), 427 cases in mixed dentition (68.3%), and 30 cases in permanent dentition (4.8%).

### 2.2. Methods

The selected cases were treated by the same surgical team. CBCT (NEWTOM, Italy) examination was performed before surgery. The image data were processed by the CBCT image analysis software to obtain the multiplanar reconstruction (standard coronal plane, sagittal plane, and horizontal plane) and three-dimensional reconstruction images of the head and face. The image data of embedded mesiodens in maxilla were observed from multiple angles to clarify the relationship between the axial position of embedded mesiodens and the adjacent normal tooth or dental germ.

All the selected cases underwent extraction of mesiodens. The specific surgical methods were as follows. First, the surgical approach was determined and then the soft tissue was cut, followed by a soft tissue flap procedure. Subsequently, intraoperative positioning was performed as the local bone was removed to identify the position of mesiodens. Finally, the space between the mesiodens and alveolar bone was expanded, and the mesiodens was extracted. For children with complex mesiodens or unable to cooperate with the surgery, general anesthesia would be suggested. Osteotome, dental turbine drill, or piezosurgery could be used when necessary.

The position, axial angle, and surgical approach of embedded mesiodens in maxilla were described and analyzed according to preoperative CBCT images and practical operation. The erupted mesiodens of the selected cases were not included in the statistical analysis and discussion.

### 2.3. Statistical Analysis

The data were analyzed by SPSS22.0. The measurement data were presented as (^−^*x* ± *s*), and the enumeration data were described as (*n*, %). The correlation between various types of embedded mesiodens and surgical approaches was analyzed by the Chi-square test. A value of *P* < 0.05 was indicative of statistical significance.

## 3. Results

There were 877 mesiodens in 625 cases, including 102 erupted mesiodens (11.6%) and 775 embedded mesiodens (88.4%) (Table 1). Specifically, 371 children (59.4%) had one mesiodens, 252 children (40.3%) had two mesiodens, of which 102 children (16.3%) had one mesiodens erupted and the other one did not erupt, and 2 children (0.3%) had three or more mesiodens (Table 2).

The axial angle of embedded mesiodens was examined by CBCT before surgery. The results demonstrated that the major axis of 120 embedded mesiodens (15.5%) presented an acute angle (including the same direction) with the major axis of adjacent normal teeth (such as 11, 21, 51, and 61); 108 embedded mesiodens (13.9%) were perpendicular to the major axis of adjacent normal teeth; 144 embedded mesiodens (18.6%) showed an obtuse angle with the major axis of adjacent normal teeth, and 403 embedded mesiodens (52.0%) were inverted (Table 3). Hence, all the selected cases were divided into five types: 82 cases of acute angle type (including the same direction) (13.1%), 68 cases of vertical type (10.9%), 118 cases of obtuse angle type (18.9%), 269 cases of inverted type (43.0%), and 88 cases of mixed type (14.1%) (Table 4).

Embedded mesiodens in maxilla of the selected cases were accurately located, and the appropriate surgical approach was adopted for extraction: 409 cases receiving the palatal gingival margin approach (PGMA) (65.4%), 100 cases receiving the combined labial-palatal approach (CLPA) (16.0%), 59 cases receiving the local palatal approach (LPA) (9.4%), 41 cases receiving the labial gingival margin approach (LGMA) (6.6%), and 16 cases receiving the local labial approach (LLA) (2.6%) (Table 5). According to statistics, the PGMA was the most selected surgical approach for the cases of acute angle (including the same direction) type, obtuse angle type, and inverted type. The PGMA and the CLPA were the most selected surgical approaches for the cases of vertical and mixed types. The Chi-square test indicated that different types of cases had different surgical approaches (*χ*^2^ = 134.203, *P* < 0.001), and there was a correlation between the type and the surgical approach of case (Cramer's *V* = 0.232, *P* < 0.001) (Table 6).

## 4. Discussion

Usually, mesiodens have no obvious symptom and only manifest as an increase in the number of teeth, but they can cause a range of complications, including tooth impaction, delayed eruption of teeth, ectopic eruption of teeth, excessive diastema of anterior teeth, malocclusion, root deformity, occlusal trauma, and odontogenic cyst [14–17]. Extraction is the main treatment of mesiodens, and the timing of extraction should be personalized according to the characteristics of each case. The prognosis is excellent after extraction and postoperative orthodontic treatment [4, 11, 18, 19].

Traditional X-ray examination, such as periapical radiography, lateral tomography, and pantomography, has played an important role in the diagnosis and treatment of mesiodens in the past. However, due to the inability to accurately locate mesiodens, it cannot meet the requirements of mesiodens extraction under the increasingly precise treatment mode [4, 20]. CBCT can determine the three-dimensional position, shape, and axial angle of mesiodens, as well as their relationship with adjacent tissues. Hence, CBCT has gradually become an essential preoperative examination for mesiodens extraction [21, 22].

Through preoperative CBCT examination, the surgeon can assess the characteristics of mesiodens accurately and thereby design an appropriate surgical approach. Different surgical approaches have their own advantages, disadvantages, and applicable conditions.

**Figure 1:**
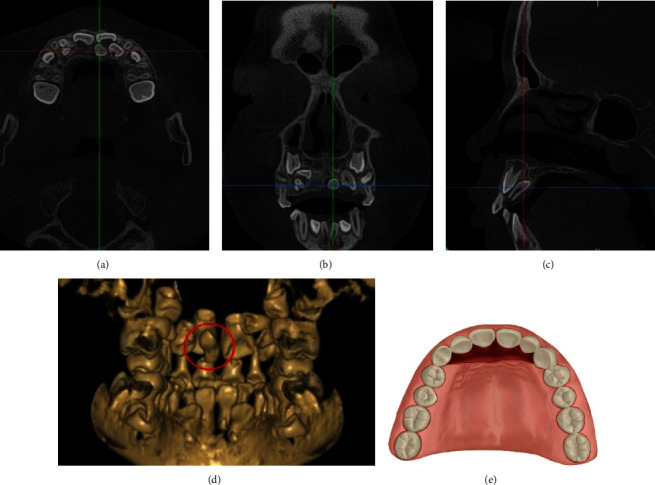
CBCT images of a typical case and the schematic plot of PGMA. (a) The horizontal plane image. (b) The coronal plane image. (c) The sagittal plane image. (d) The three-dimensional reconstruction image. (e) The schematic plot of simulated incision of PGMA.

The palatal gingival margin approach (PGMA) is the most common surgical approach in the extraction of mesiodens in maxilla in children. The applicable conditions include the major axis of the mesiodens is basically parallel to the major axis of the adjacent normal deciduous tooth or permanent tooth (that is, the direction of the crown of the mesiodens is consistent with that of the adjacent normal deciduous tooth or permanent tooth, or the mesiodens is inverted); the mesiodens is generally located on the palatal side and close to the palatal osteone and alveolar process, and there is no need for an excess of flap surgery and bone removal. The advantages include obtaining better surgical field, easy positioning for mesiodens, and less cicatricial tissue after surgery. The disadvantage is that the gingival papilla or incisiva papillae may be damaged during flap surgery (Figure 1).

**Figure 2:**
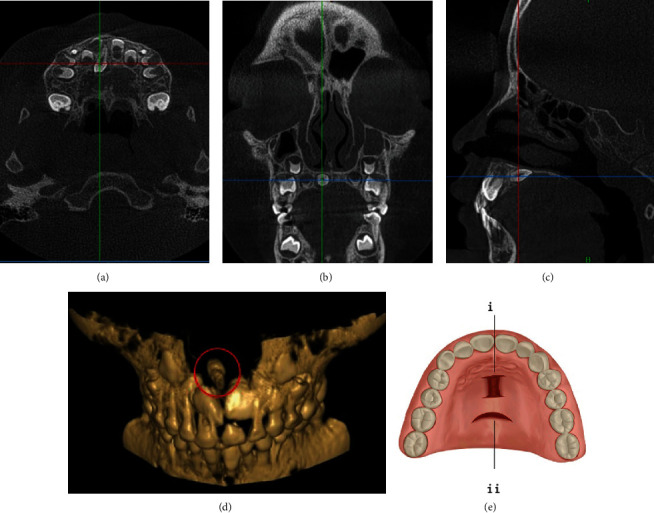
CBCT images of a typical case and the schematic plot of LPA. (a) The horizontal plane image. (b) The coronal plane image. (c) The sagittal plane image. (d) The three-dimensional reconstruction image. (e) The schematic plot of simulated incision of (i) the palatal I-type approach and (ii) the palatal arcuate approach.

The local palatal approach (LPA) includes but is not limited to the palatal arcuate approach and the palatal I-type approach. When the major axis of the mesiodens is basically parallel to the major axis of the adjacent normal deciduous tooth or permanent tooth (that is, the direction of the crown of the mesiodens is consistent with that of the adjacent normal deciduous tooth or permanent tooth, or the mesiodens is inverted), moreover, the mesiodens is far from the alveolar process, and especially the mesiodens is located at the horizontal of the hard palate, that is, the applicable condition. The advantages include less tissue damage, less bleeding in the surgical area, and smaller range of flap surgery. The disadvantages include smaller surgical field, more precise location of the mesiodens, and easier formation of cicatrice after surgery (Figure 2).

**Figure 3:**
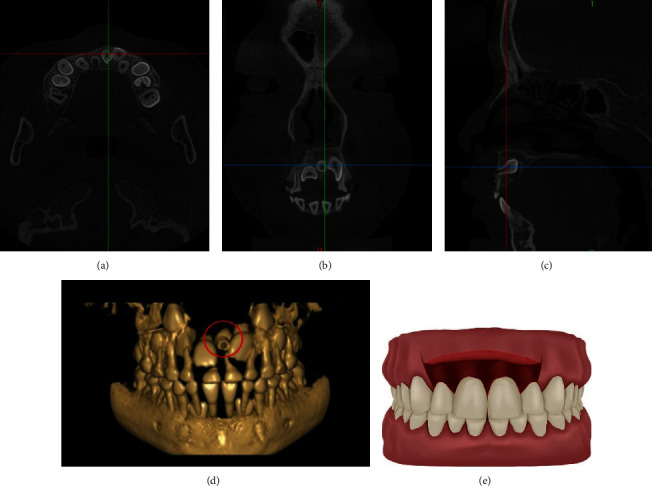
CBCT images of a typical case and the schematic plot of LGMA. (a) The horizontal plane image. (b) The coronal plane image. (c) The sagittal plane image. (d) The three-dimensional reconstruction image. (e) The schematic plot of simulated incision of LGMA.

The labial gingival margin approach (LGMA) can be performed when the crown or root of the mesiodens is close to the labial osteone because it is relatively rare for mesiodens to be located on the labial side of maxilla. By this approach, surgeon can get larger surgical field and fewer cicatrices after surgery. Though it is easier to damage the adjacent normal teeth during bone removal and lead to gingival papilla laceration during flap surgery. As an auxiliary incision, the angular or trapezoidal incision can be made at the same time to reduce the range of flap. But it is necessary to weigh the pros and cons and make a choice according to the specific situation because of formation of cicatrice in the labial gingival region and having influence on the appearance (Figure 3).

**Figure 4:**
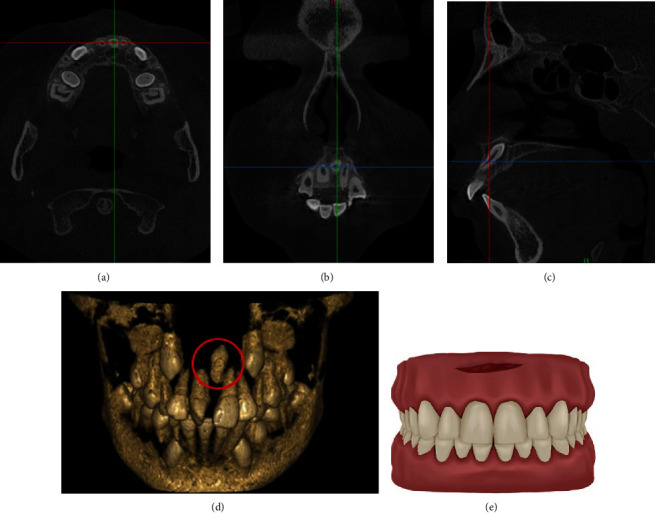
CBCT images of a typical case and the schematic plot of LLA. (a) The horizontal plane image. (b) The coronal plane image. (c) The sagittal plane image. (d) The three-dimensional reconstruction image. (e) The schematic plot of simulated incision of LLA.

The local labial approach (LLA) can be performed at the labial vestibular groove when the crown or root of mesiodens is near the labial side, but the position is far from the alveolar process, or even close to the nasal base. It should be noted that the surgical incision should keep away from the attached gingiva. The advantages include getting closer to mesiodens and bringing about less tissue damage. The disadvantages include giving rise to normal teeth damage because of the adjacent relation between mesiodens and normal teeth, more bleeding in the surgical area, soft tissue edema, and formation of cicatrice after surgery (Figure 4).

**Figure 5:**
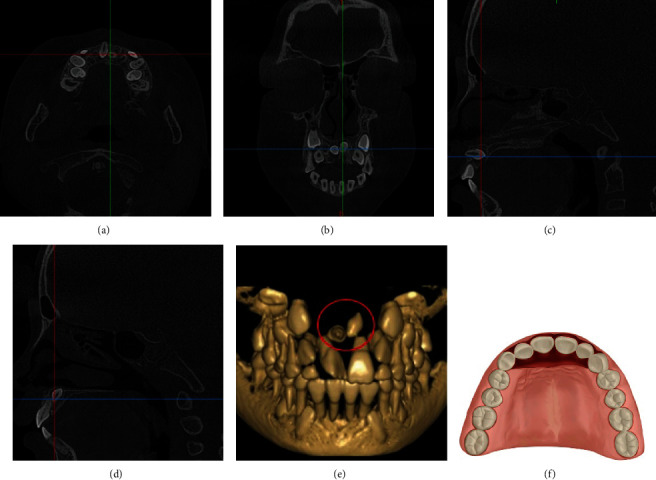
CBCT images of a typical case and the schematic plot of CLPA. (a) The horizontal plane image. (b) The coronal plane image. (c, d) The sagittal plane images. (e) The three-dimensional reconstruction image. (f) The schematic plot of simulated incision of CLPA.

The combined labial-palatal approach (CLPA) should be considered before surgery when the major axis of the mesiodens is perpendicular or nearly perpendicular to the major axis of the normal adjacent deciduous tooth or permanent tooth, that is, the crown of the mesiodens faces the labial or palatal side. Moreover, if the mesiodens cannot be removed by a unilateral (palatal or labial) approach during the surgery, the combined labial-palatal approach should also be considered. Hence, having a sufficient surgical field is the advantage, and if necessary, the mesiodens can be taken out from the crown side by tapping the root side of the mesiodens. Furthermore, getting larger tissue damage and more bleeding in the surgical area are the disadvantages (Figure 5).

**Figure 6:**
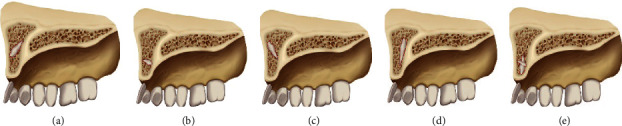
Classification of the cases in our study. (a) The acute angle type (including the same direction). (b) The vertical type. (c) The obtuse angle type. (d) The inverted type. (e) The mixed type.

An appropriate surgical approach can facilitate the extraction of mesiodens. The determination of surgical approach before surgery should keep to three following principles: first, the shortest linear distance to mesiodens, second, the least tissue damage, and third, the best protection of adjacent teeth and tooth germs. Therefore, on basis of these three principles and CBCT images of cases, we summarized the selection method of surgical approach for mesiodens in maxilla in children. According to the relationship in sagittal plane between the major axis of the crown of mesiodens and the major axis of the crown of adjacent normal teeth (deciduous teeth or permanent teeth), cases were classified into the acute angle type (including the same direction), the vertical type, the obtuse angle type, the inverted type, and the mixed type (Figure 6).

The acute angle type (including the same direction): priority should be given to the gingival margin approach on the side where the crown of mesiodens is closer to the osteone; if the mesiodens is near the nasal floor or the horizontal of the hard palate, the local approach on this side (labial vestibular groove approach or palatal arc approach or palatal I-type approach) can be considered.

The vertical type: priority should be given to the gingival margin approach on the side of the crown or root of mesiodens closer to the osteone; if the distance from the mesiodens to the labial and palatal osteones is similar, the gingival margin approach on the crown side is preferred; if the mesiodens is close to the horizontal of the hard palate, the local palatal approach (arc approach or I-type approach) is preferred; if the structure of the mesiodens is complex (e.g., the root is S-shaped) or has fetters with the surrounding tissues, the combined labial-palatal approach can be considered.

The obtuse angle type: priority should be given to the gingival margin approach on the side of the mesiodens (crown or root) closer to the osteone and closer to the alveolar process; if the mesiodens is near the nasal base or the horizontal of the hard palate, the local approach on this side (labial vestibular groove approach or palatal arc approach or palatal I-type approach) can be considered.

The inverted type: priority should be given to the gingival margin approach on the side where the root of mesiodens is closer to the osteone; if the distance from the mesiodens to the labial and palatal osteones is similar, the palatal gingival margin approach is preferred; if the mesiodens is near the nasal base or the horizontal of the hard palate, the local approach on this side (labial vestibular groove approach or palatal arc approach or palatal I-type approach) can be considered.

The mixed type: if there are several mesiodens and their positions differ greatly, the extraction strategy of the more complex one should be fully considered and the surgical approach should be designed reasonably, and the extraction of the simpler one can be given priority during the surgery.

As for special cases, the surgical approach should be flexibly selected according to the actual situation. For instance, if the mesiodens is located at the nasal base, the transnasal approach or the labial vestibular groove-nasal base flap approach can be considered; if the mesiodens is complicated by the odontogenic cyst, the approach from the weak side of the bone (gingival margin approach or local approach) can be considered; and if there is a corresponding axial relationship between the mesiodens and the adjacent normal teeth (deciduous teeth or permanent teeth) in coronal plane, a gingival margin or local approach on the side of the mesiodens with tissue closer to the bone plate can be considered.

To conclude, the method of selecting the surgical approach presented in this study may not be applicable to all possible cases of maxillary embedded mesiodens in children. In clinical work, it is noted that approaches do vary from one another, and surgeons need to fully study the image data and choose the most appropriate surgical approach for children according to the specific location of mesiodens in maxilla.

## Figures and Tables

**Table 1:** The state of eruption of all mesiodens.

State	Erupted	Embedded	Total
Number of mesiodens	102	775	877
Proportion (%)	11.6	88.4	100

**Table 2:** The amount of mesiodens of each case.

Number of mesiodens	1 embedded	2 mesiodens		3 or more	Total
		1 erupted, 1 embedded	2 embedded		
Number of cases	371	102	150	2	625
Proportion (%)	59.4	16.3	24	0.3	100

**Table 3:** The axial angle of embedded mesiodens.

Axial angle	Acute (including the same direction)	Vertical	Obtuse	Inverted	Total
Number of embedded mesiodens	12	108	144	403	775
Proportion (%)	15.5	13.9	18.6	52	100

**Table 4:** The type of selected cases.

Type	Acute (including the same direction)	Vertical	Obtuse	Inverted	Mixed	Total
Number of cases	82	68	118	269	88	625
Proportion (%)	13.1	10.9	18.9	43	14.1	100

**Table 5:** The surgical approach of selected cases.

Approach	PGMA	LPA	LGMA	LLA	CLPA	Total
Number of cases	409	59	41	16	100	625
Proportion (%)	65.4	9.4	6.6	2.6	16	100

PGMA: palatal gingival margin approach; LPA: local palatal approach; LGMA: labial gingival margin approach; LLA: local labial approach; CLPA: combined labial-palatal approach.

**Table 6:** The relationship between the type of the cases and surgical approach.

Type of cases	Type of approach, number of cases (residuals)				
	PGMA	LPA	LGMA	LLA	CLPA
Acute (including the same direction)	50 (-0.9)	6 (-0.7)	9 (1.7)	4 (1.4)	13 (0)
Vertical	26 (-5)	8 (0.7)	7 (1.3)	5 (2.7)	22 (3.9)
Obtuse	65 (-2.6)	24 (4.5)	14 (2.6)	3 (0)	12 (-1.9)
Inverted	225 (8.3)	15 (-2.9)	9 (-2.8)	2 (-2.5)	18 (-5.5)
Mixed	43 (-3.5)	6 (-0.9)	2 (-1.8)	2 (-0.2)	35 (6.6)

*χ* ^2^ = 134.203, *P* < 0.001. Cramer's *V* = 0.232, *P* < 0.001. PGMA: palatal gingival margin approach; LPA: local palatal approach; LGMA: labial gingival margin approach; LLA: local labial approach; CLPA: combined labial-palatal approach.

## Data Availability

The datasets used to support the study are available from the corresponding author on reasonable request.
